# Detection of Sporadic Outbreaks of Rift Valley Fever in Uganda through the National Viral Hemorrhagic Fever Surveillance System, 2017–2020

**DOI:** 10.4269/ajtmh.22-0410

**Published:** 2023-03-13

**Authors:** Luke Nyakarahuka, Shannon Whitmer, John Klena, Stephen Balinandi, Emir Talundzic, Alex Tumusiime, Jackson Kyondo, Sophia Mulei, Ketan Patel, Jimmy Baluku, Gloria Akurut, Diana Namanya, Kilama Kamugisha, Caitlin Cossaboom, Amy Whitesell, Carson Telford, James Graziano, Joel Montgomery, Stuart Nichol, Julius Lutwama, Trevor Shoemaker

**Affiliations:** 1 Department of Arbovirology, Emerging and Reemerging Infectious Diseases, Uganda Virus Research Institute, Entebbe, Uganda;; 2 Department of Biosecurity, Ecosystems and Veterinary Public Health, Makerere University, Kampala, Uganda;; 3 Viral Special Pathogens Branch, Division of High-Consequence Pathogens and Pathology, U.S. Centers for Disease Control and Prevention, Atlanta, Georgia;; 4 Uganda Wildlife Authority, Kampala, Uganda

## Abstract

Rift Valley fever (RVF) is a zoonotic disease of public health and economic importance. Uganda has reported sporadic outbreaks of RVF in both humans and animals across the country, especially in the southwestern part of the “cattle corridor” through an established viral hemorrhagic fever surveillance system. We report 52 human cases of laboratory-confirmed RVF from 2017 to 2020. The case fatality rate was 42%. Among those infected, 92% were males and 90% were adults (≥ 18 years). Clinical symptoms were characterized by fever (69%), unexplained bleeding (69%), headache (51%), abdominal pain (49%), and nausea and vomiting (46%). Most of the cases (95%) originated from central and western districts that are part of the cattle corridor of Uganda, where the main risk factor was direct contact with livestock (*P* = 0.009). Other predictors of RVF positivity were determined to be male gender (*P* = 0.001) and being a butcher (*P* = 0.04). Next-generation sequencing identified the predominant Ugandan clade as Kenya-2, observed previously across East Africa. There is need for further investigation and research into the effect and spread of this neglected tropical disease in Uganda and the rest of Africa. Control measures such as promoting vaccination and limiting animal–human transmission could be explored to reduce the impact of RVF in Uganda and globally.

## INTRODUCTION

Rift Valley fever (RVF) is a zoonotic viral hemorrhagic fever (VHF) caused by infection with Rift Valley fever virus (RVFV), a negative-sense enveloped RNA virus in genus *Phlebovirus* and the recently reclassified family Phenuiviridae.^1^ Rift Valley fever virus was first described in the early 1900s in the Rift Valley region of Kenya when it caused outbreaks in livestock. The virus has caused outbreaks both in animals and humans mainly in East Africa, but it has also been reported in Sudan, Saudi Arabia, and Yemen.^2^ In 2016, Uganda reported the first outbreak of RVF since 1963, when RVFV was confirmed in samples from three humans and one goat from the southwestern district of Kabale.^3^ Rift Valley fever is a disease of public health and economic importance, affecting humans and livestock as well as international trade. It affects livestock production by reducing milk yield and causing abortions; however, most animals do not have obvious clinical symptoms. Also, most human infections do not exhibit severe clinical signs or symptoms. Documented clinical symptoms in humans include fever, headache, nausea, musculoskeletal pain, diarrhea, vomiting, cough, and bleeding from body orifices, characteristic of severe forms of VHFs.^4^

Rift Valley fever virus is diagnosed by the detection of viral RNA in the blood of infected persons, animals, or mosquitoes by reverse transcription–quantitative polymerase chain reaction (RT-qPCR), but anti-RVFV antibodies indicating recent or past infection can also be detected by serological approaches such as ELISA.^5^

Rift Valley fever virus is transmitted predominantly between animals and humans by *Aedes *spp. and *Culex *spp. Mosquitoes, which have been described as competent vectors. In Uganda, RVFV RNA has been detected in both mosquito genera.^3^ Other mosquito species may also be involved in the transmission of RVFV, and mechanical transmission of RVFV has been documented for hematophagous dipterans and other insect vectors.^6^ Humans are infected most often through contact with infected livestock or their by-products.^7^ A study conducted in Uganda^8^ found that abattoir workers who are regularly in contact with livestock body fluids were at significant risk of seropositivity to RVFV. That study also reported an RVFV seroprevalence of 27% in cattle and 13% in humans.^8^ Similar studies in other countries also show that risk for human infections is mainly from contact with livestock, but transmission can occur by mosquito bite.^9^^,^^10^ Risk groups include occupations associated with farming, such as veterinarians, herdsmen, and farmers who cultivate the bush, where they interact with mosquito vectors with zoophilic potential.^11^ Person-to-person transmission of RVFV has not been documented.^12^

Since 2010, Uganda has had the capacity to detect RVF infections, together with other VHFs, at the Uganda Virus Research Institute (UVRI) through the National Viral Hemorrhagic Fever Surveillance Program, where samples from humans with suspect VHFs are submitted and tested routinely.^13^ After the detection of three human cases reported in Kabale in 2016,^3^ Uganda has continued to report an increase in sporadic outbreaks of human RVF with associated mortality. We describe the emerging epizootic of RVF human cases reported from 2017 to 2020 as documented through the national VHF surveillance system, focusing on the epidemiological and laboratory characteristics and findings.

## METHODS

### Case Reporting.

Rift Valley fever suspect cases are reported through the national VHF surveillance system coordinated through UVRI and the Ministry of Health using a VHF suspect case definition as described previously.^13^ This is achieved by both passive and active surveillance approaches coupled with a sentinel VHF surveillance system. Viral hemorrhagic fever case definitions are distributed throughout health facilities in Uganda according to the Integrated Disease Surveillance and Response guidelines, through the VHF surveillance system, and via VHF sentinel surveillance sites. The suspect case definition is a patient with acute illness, fever > 38°C, no clear alternative diagnosis, and at least four of the following signs or symptoms: vomiting/nausea, diarrhea, muscle or joint pain, chills, rigors, intense fatigue, abdominal pain, skin rash, difficulty in swallowing, headache, or unexplained bleeding from any site.

Physicians assess patients presenting at health facilities for suspected VHF. If the patients meet the VHF case definition, a blood sample is collected according to internationally recognized biosafety and biosecurity measures.^14^ During outbreaks, individuals are also included if they have high-risk contact with a confirmed case or a high-risk exposure, regardless of whether they meet the case definition. When identified, individuals with suspect infection are isolated. To collect blood samples, physicians and laboratory technicians don appropriate personal protective equipment (PPE). The official Uganda VHF Case Investigation Form is completed for every suspect case and it accompanies the sample. Samples are triple-packaged and transported to the VHF referral laboratory at UVRI Entebbe via the national sample transport system.^15^ Upon arrival at UVRI, samples are assessed for quality and appropriateness. RNA is extracted and tested for the presence of VHFs, including RVFV, by RT-qPCR.^16^ For some samples, IgM and IgG antibodies targeting RVFV are detected by ELISA using methods described previously.^2^^,^^3^^,^^5^

### Confirmed Case Investigations.

The UVRI VHF program staff investigates all confirmed human cases to understand the epidemiology of the diseases more fully. For RVF, investigations also focus on identifying the possible source of infection (e.g., from a mosquito vector, direct contact with infected livestock or their body fluids). Case investigation questionnaires are administered to patients or their attendants. Also, geographic coordinates are collected to map the location of cases and, later, to study the environment where the patients are living. For homesteads with livestock residing near patients, animal samples are collected according to zoonotic disease outbreak investigations, as available, to assess potential zoonotic transmission. During field investigations, health education and outreach are conducted, because communities want and need to understand aspects of the epidemiology of RVF, and control and prevention strategies are discussed as well. Health education posters are supplied that are designed to provide information based on data and observations from previous investigations and knowledge assessments.^17^ These activities were reviewed by the CDC and were conducted consistent with applicable federal law and CDC policy.^1^

### Data Management.

Epidemiological data was managed using the EpiInfo database^18^ and exported to R-studio (R Foundation for Statistical Computing, Vienna, Austria) for additional analysis. Data were analyzed for bivariate associations using the χ^2^ test for normally distributed variables or Fisher’s exact test for nonparametric variables. The logistic regression model was used to assess for risk factors. We compared RVF confirmed cases with suspect individuals who tested negative for RVF. Those who tested negative were either family members of the confirmed cases or individuals who lived in the same area as confirmed cases with the same exposures.

### RNA Extraction, NGS Library Preparation, bioinformatics and phylogenetics.

RNA was extracted from blood or serum specimens with Tripure and Zymo RNA Clean and Concentrator-25 kits at the BSL-4 laboratory at the U.S. CDC or with MagMax in the High Containment Laboratory at UVRI, Uganda. RNA was DNase-treated and NGS libraries were made using the NEBNext Ultra II Directional RNA library prep kit. Libraries were pooled to low plexy (three- to six-plex) or were run individually on a MiSeq v2 300 cycle, MiniSeq High output 300 cycle, or iSeq 300 cycle cartridge. Initial RVFV genomes (20190877, 20190879, and 20190880) were assembled using a guided de novo approach with viral-ngs (version 1.22.1; Broad Institute) and a custom-made RVFV-specific lastal database. The 201902760 genome was built by mapping reads to the 201900879 reference genome using in-house scripts [Illumina index removal with cutadapt, quality trim reads with prinseq-lite (-min_qual_mean 25 -trim_qual_right 20 -min_len 50), mapped to reference with minimap2, removal of PCR duplicates with picard MarkDuplicates and consensus genomes called with Geneious (threshold 0%, majority; assign quality = Total; call N if coverage < 2)]. All other consensus genomes were constructed using 1) de novo assembly and blasting of contigs to identify the closest reference sequence in GenBank, followed by iterative mapping of reads and contigs to the closest reference sequence; and 2) iterative mapping of reads and contigs to 201900879 and 201902760 genomes, which are representative members of two separate RVFV clades in Uganda. Consensus genomes were called from bam files with the best read coverage using iVar (version 1.3, -m 2 -n N).^19^ For two genomes (201902733 and 201902754), mapping of reads and contigs introduced large indels or frameshifts. In these cases, reads were remapped to the closest matching reference. To build phylogenetic trees, all available full-length S, M, and L RVFV segments were downloaded from GenBank and aligned using MAFFT (version 7.450), and trees were constructed with RAxML (version 7.3.0, -m GTRGAMMA -p $RANDOM -f a -x $RANDOM -N 1000). Trees were visualized with ggtree and clades were labeled according to Grobbelaar et al.^20^ and Samy et al.^21^ GenBank accession no. are ON060771 to 838.

## RESULTS

### Epidemiological Description of the Cases.

**Figure 1.**
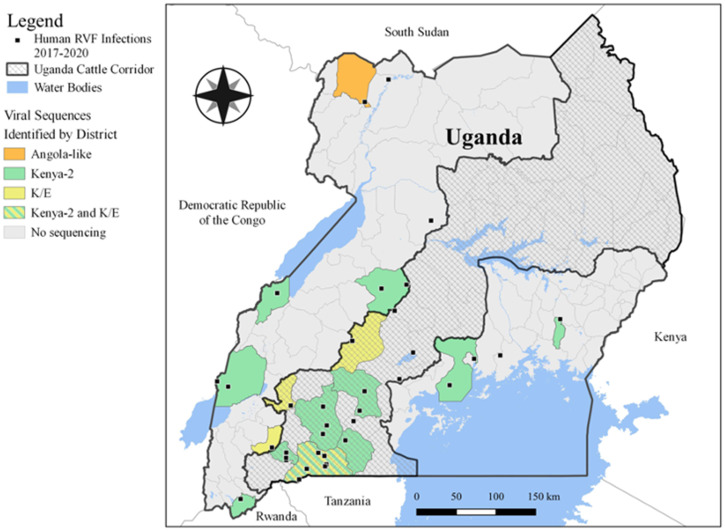
Map of Uganda showing the location of Rift Valley fever (RVF)–confirmed human cases, cattle corridor of Uganda, and physical features. K/E = K/E clade.

**Figure 2.**
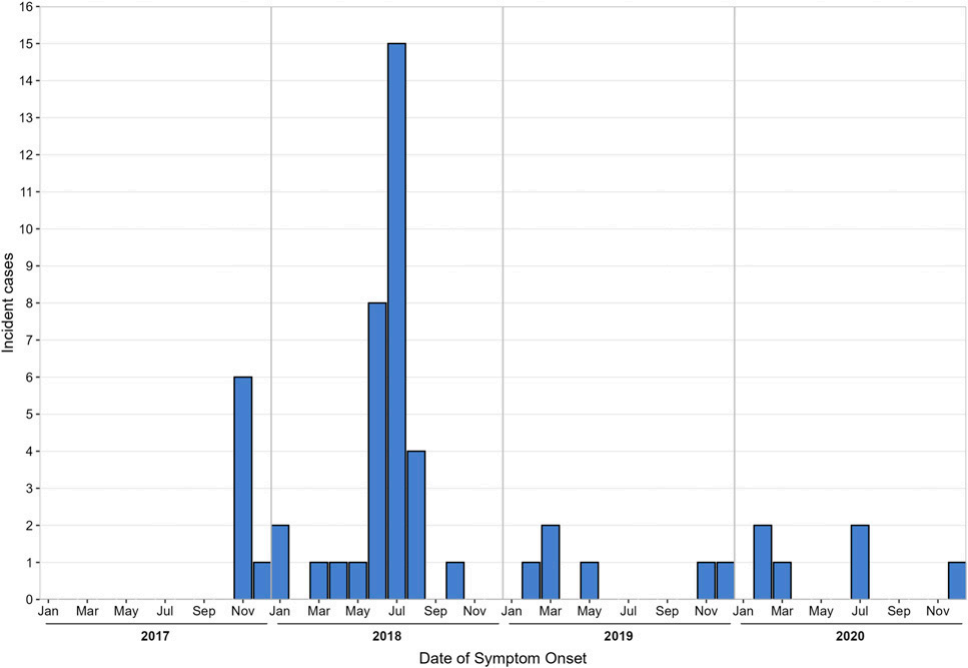
Confirmed cases of Rift Valley fever in Uganda from 2017 to 2020.

From January 2017 to December 2020, RVFV was confirmed in 52 cases using either RT-qPCR (*n* = 37) or ELISA (*n* = 15). Thirty-eight cases (73%) were detected through passive surveillance, whereas 14 cases (27%) were detected through outbreak investigations (Table 1). The mean age of the cases was 31.6 years (SD = 15.5 years), with an age range of 10 to 69 years, although adults ≥ 18 years represented 90% of cases. Ninety-two percent of cases (48 of 52) were male. Almost half the confirmed cases died, representing a case fatality rate (CFR) of 42% (22 of 52). Most of the patients (55%) reported being in contact with livestock either through tending to them as herdsmen or through slaughter. The largest proportion of cases occurred in districts inside the “cattle corridor” of Uganda (67%, *n* = 36), especially in southwestern Uganda, followed by districts in Central Uganda (25%, *n* = 13). Individual cases were also identified in the northern districts of Yumbe and Obongi, and the eastern district of Iganga (Figure 1). Most of the patients had fever (69%) and unexplained bleeding (69%) (Table 2). Other symptoms included diarrhea (38%), abdominal pain (49%), intense fatigue (61%), headache (51%), and anorexia (61%). Although outbreaks seemed to follow a seasonal pattern, following the heavy rains of March, April, and May in year 2018, they did not follow a similar pattern for the rest of the years (Figure 2). However, it is important to note that 2018 had heightened surveillance because of Ebola virus disease (EVD) outbreak in the neighboring Democratic Republic of the Congo (DRC).^22^

**Table 1:** Sociodemographic variables for RVF cases (*N* = 52) detected in Uganda, 2017 to 2020

Characteristic	*n* (%)
Status	
Alive	30 (58)
Dead	22 (42)
Surveillance method	
Active	14 (27)
Passive	38 (73)
Age, years	
< 18	5 (10)
≥ 18	45 (90)
Gender	
Female	4 (7.7)
Male	48 (92)
Region of Uganda	
East	3 (5.8)
West	36 (69)
Central	13 (25)
Farmer occupation	23 (44)
Butcher occupation	9 (17)
Travel history	4 (10)
Contact with animals	22 (55)

RVF = Rift Valley fever.

**Table 2:** Clinical symptoms of the patients confirmed of having RVF

Characteristic	% (*n*/N*)
Fever present	69 (31/45)
Unexplained bleeding	69 (31/45)
Intense fatigue	61 (23/38)
Anorexia	61 (22/36)
Abdominal pain	49 (19/39)
Headache	51 (19/37)
Vomiting and nausea	46 (18/39)
Diarrhea	38 (15/40)
Muscle pain	36 (13/36)
Joint pain	32 (12/37)
Jaundice	31 (11/35)
Chest pain	28 (10/36)
Conjunctivitis	26 (9/34)
Sore throat	24 (8/34)
Difficulty breathing	19 (7/36)
Hiccups	15 (5/34)
Cough	11 (4/35)
Skin rash	3.0 (1/33)

RVF = Rift Valley fever.

* Denominator (*N*) indicates records with complete data in the respective variable. Missing records were dropped during analysis.

When comparing individuals who tested positive for RVFV with those who tested negative from the same homesteads, statistically significant relationships were found for most sociodemographic factors (Table 3). Bivariate analysis using a χ^2^ test found that RVF positivity was associated with age, gender, geographic region of Uganda, being a butcher, and having contacts with livestock (Table 3). Multivariate logistic regression found significant predictors of RVF positivity: male gender (*P* = 0.001), being a butcher (*P* = 0.04), and having direct animal contact (*P* = 0.03) (Table 4).

**Table 3:** Bivariate analysis assessing the association between RVF positivity and sociodemographic factors

Characteristic	Overall (*N* = 146), *n *(%)	RVF negative (*n* = 94), *n *(%)	RVF positive (*n* = 52), *n *(%)	*P* value
Status				
Alive	113 (77)	83 (88)	30 (58)	**< 0.001**
Dead	33 (23)	11 (12)	22 (42)	
Surveillance method				
Active	14 (10)	0 (0)	14 (27)	**< 0.001**
Passive	132 (90)	94 (100)	38 (73)	
Age, years				
< 18	27 (19)	22 (23)	5 (10)	**0.050**
≥ 18	117 (81)	72 (77)	45 (90)	
Gender				
Female	38 (26)	34 (36)	4 (7.7)	**< 0.001**
Male	108 (74)	60 (64)	48 (92)	
Region				
East	3 (2.1)	0 (0)	3 (5.8)	**0.032**
West	113 (77)	77 (82)	36 (69)	
Central	30 (21)	17 (18)	13 (25)	
Farmer	52 (36)	29 (31)	23 (44)	0.11
Butcher	11 (7.5)	2 (2.1)	9 (17)	**0.002**
Animal contact	48 (38)	26 (31)	22 (55)	**0.009**

RVF = Rift Valley fever. *P* values in bold type are significant.

**Table 4:** Multivariate logistic regression model of sociodemographic factors as predictors of RVF positivity in humans

Characteristic	OR	95% CI	*P* value
Status			
Alive	Ref	–	
Dead	16.0	4.41–73.2	**< 0.001**
Age, years			
< 18	Ref	–	
≥ 18	2.52	0.50–19.3	0.3
Gender			
Female	Ref	–	
Male	59.0	7.89–1,454	**< 0.001**
Farmer			
No	Ref	–	
Yes	1.63	0.50–5.35	0.4
Butcher			
No	Ref	–	
Yes	32.1	2.22–1,935	**0.041**
Travel history			
No	Ref	–	
Yes	1.21	0.17–7.06	0.8
Animal contact			
No	Ref	–	
Yes	3.77	1.14–13.9	**0.035**

OR = odds ratio; Ref = reference; RVF = Rift Valley fever. *P* values in bold type are significant.

We assessed clinical symptoms associated with RVF positivity (Table 5). In bivariate analyses, only cough (*P* = 0.03) and jaundice (*P* = 0.02) were associated with RVF positivity. However, the multivariate logistic regression analysis revealed that RVF positivity was best predicted by clinical symptoms of fever, anorexia, intense fatigue, chest pain, cough, sore throat, and conjunctivitis (Table 6).

**Table 5:** Bivariate analysis assessing RVF positivity and clinical symptoms

Characteristic	*n*	RVF test result			*P* value†
		Overall (*N* = 146),* n *(%)	Negative (N = 94), % (*n*/N*)	Positive (*n* = 52), % (*n*/N*)	
Fever	109	81 (74)	78 (50/64)	69 (31/45)	0.3
Unexplained bleeding	118	75 (64)	60 (44/73)	69 (31/45)	0.3
Vomiting and nausea	106	52 (49)	51 (34/67)	46 (18/39)	0.6
Diarrhea	102	41 (40)	42 (26/62)	38 (15/40)	0.7
Intense fatigue	98	64 (65)	68 (41/60)	61 (23/38)	0.4
Anorexia	95	49 (52)	46 (27/59)	61 (22/36)	0.15
Abdominal pain	104	58 (56)	60 (39/65)	49 (19/39)	0.3
Chest pain	90	27 (30)	31 (17/54	28 (10/36)	0.7
Muscle pain	100	42 (42)	45 (29/64)	36 (13/36)	0.4
Joint pain	91	33 (36)	39 (21/54)	32 (12/37)	0.5
Headache	101	57 (56)	59 (38/64)	51 (19/37)	0.4
Cough	89	21 (24)	31 (17/54)	11 (4/35)	**0.030**
Difficulty breathing	92	13 (14)	11 (6/56)	19 (7/36)	0.4
Sore throat	93	18 (19)	17 (10/59)	24 (8/34)	0.4
Jaundice	85	17 (20)	12 (6/50)	31 (11/35)	**0.028**
Conjunctivitis	88	18 (20)	17 (9/54)	26 (9/34)	0.3
Skin rash	89	6 (6.7)	9 (5/56)	3 (1/33)	0.4
Hiccups	88	7 (8.0)	3.7 (2/54)	15 (5/34)	0.10

RVF = Rift Valley fever.* P* values in bold type are significant.

* Denominators were computed only for variables with complete data. Missing data records were dropped.

† Pearson’s χ^2^ test for normally distributed variables; Fisher’s exact test for non-normally distributed variables.

**Table 6:** Multivariate logistic regression model of clinical symptoms as predictors of RVF positivity in humans

Characteristic	OR	95% CI	*P* value
Fever			
No	Ref	–	
Yes	0.26	0.07–0.86	**0.033**
Intense fatigue			
No	Ref	–	
Yes	0.35	0.06–1.62	0.2
Anorexia			
No	Ref	–	
Yes	4.56	1.02–26.4	0.061
Chest pain			
No	Ref	–	
Yes	4.38	1.02–21.8	0.055
Cough			
No	Ref	–	
Yes	0.34	0.06–1.50	0.2
Sore throat			
No	Ref	–	
Yes	3.27	0.69–17.5	0.14
Conjunctivitis			
No	Ref	–	
Yes	0.37	0.08–1.53	0.2

OR = odds ratio; RVF = Rift Valley fever. *P* values in bold type are significant.

**Figure 3.**
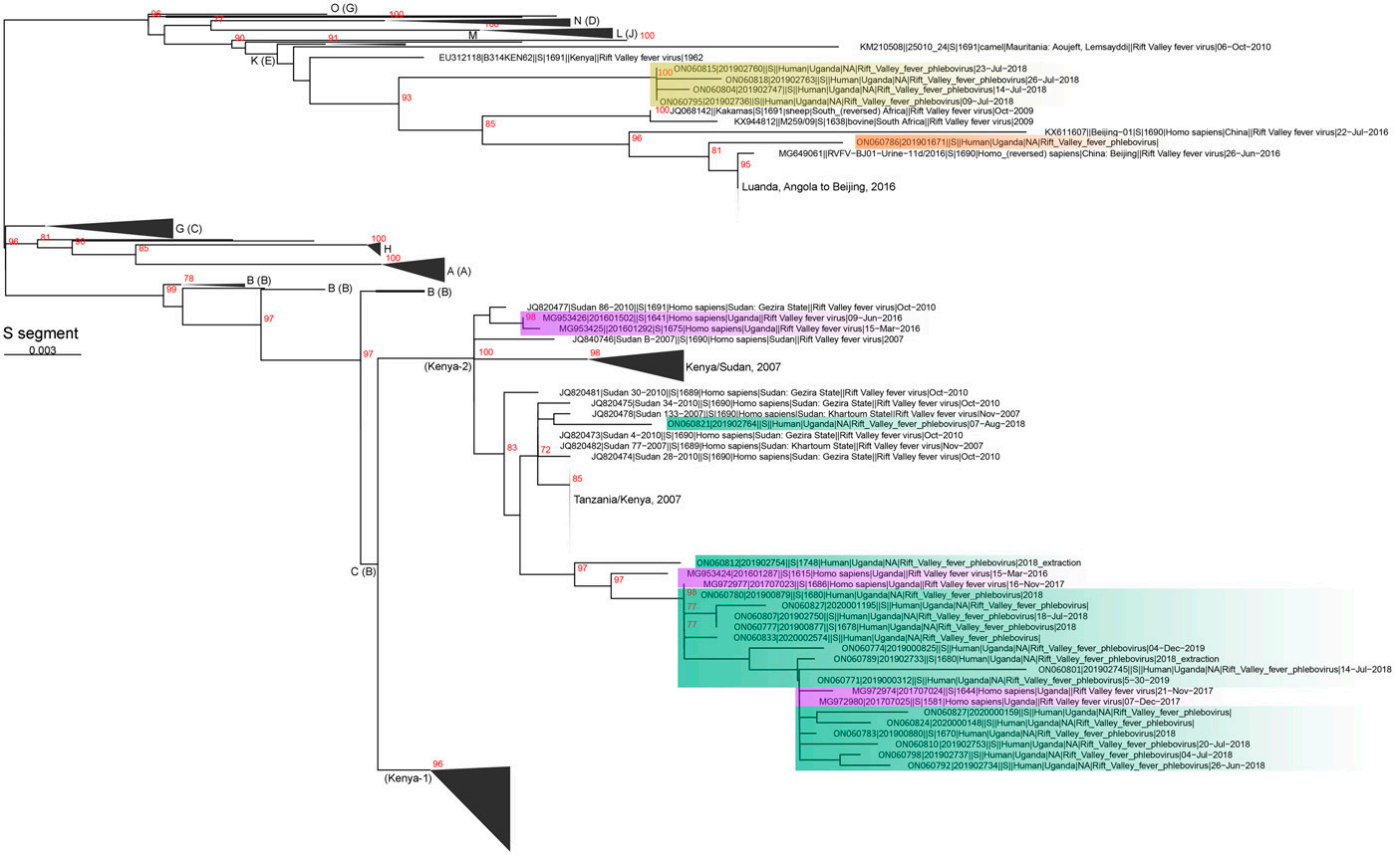
Phylogenetic tree of Rift Valley fever S segments from 2017 to 2020 for human cases. Green, yellow, and orange shading highlights new sequences according to specimen collection locations in Figure 1; purple shading highlights historical Ugandan sequences.

In Figure 3, clades are highlighted in orange, yellow, or green according to specimen collection locations in Figure 1. Major clades are labeled according to Grobbalaar et al.^20^ and Samy et al.^21^ (in parentheses). Nodes with bootstrap support > 70% are labeled in red, and the scale bar is in units of substitutions/site.

### Phylogenetic Relatedness of RVFV Strains Collected in Uganda.

Next-generation sequencing of RVFV RT-qPCR–positive specimens identified two distinct clades circulating within Uganda (Figure 3). We observed that the majority of new RVFV sequences collected between 2017 and 2020 clustered in the Kenya-2 clade. This clade is distributed broadly across East Africa and includes sequences collected from a large epizootic outbreak in Kenya in 2006 to 2007, as well as sequences collected in Sudan in 2007 and 2010, and from Uganda in 2016 to 2020. The Kenya-2 clade has been detected in both West and Central Uganda (Figures 1 and 3, green). We also noted that sequences collected from 2018 in Uganda cluster in a second clade that is distinct from Kenya-2 (Figure 3, yellow and orange). This K/E clade contains sequences collected in 2009 in South Africa, and the Ugandan sequences are either ancestral to the South African clade or a member of the clade. One Ugandan sequence collected in January 2018 from a refugee camp in the Yumbe district (northwestern Uganda) shares a most recent common ancestor with a sequence imported into Beijing from a forklift worker originally working in Luanda, Angola, in 2016^23^ (Figures 1 and 3, orange). In contrast, the ancestral Ugandan K/E clade contains four sequences collected in July 2018 from districts in southwestern Uganda: Ibanda, Mubende, Isingiro, and Sheema (Figures 1 and 3, yellow).

## DISCUSSION

Uganda reported the first contemporary case of RVF in 2016, which was the first report since 1963, when cases of RVF were detected in mosquitoes and humans in Lunyo, Entebbe.^24^ Reemergence of the virus was reported in the southwestern district of Kable in two males, both of whom survived.^3^ Detection of these cases was made possible by the establishment of a diagnostic and surveillance system for VHFs at UVRI beginning in 2010. These cases were initially suspected to be EVD or Marburg virus disease, given that the initial case presented with hemorrhagic symptoms and there had been a past outbreak of Marburg virus disease in the region.^25^ Follow-up studies showed that the prevalence of RVF in the Kabale region was ∼13% in both humans and animals, indicating the virus could have been circulating well before 2016.^8^ Because of the heightened interest in RVF in Uganda, 52 additional cases of RVF have since been confirmed (2017–2020) (Table 1). The CFR was 42% (22 of 52), which is considerably higher than that of other documented outbreaks of RVF in East Africa. For example, an outbreak in Kenya in 2006 and 2007 reported a fatality rate of 26% to 29%.^26^^,^^27^ The high CFR of RVF in Uganda reinforces the status of RVF as a severe disease that merits the attention of both national and international health surveillance systems. Because RVF typically presents as a mild febrile illness, we suspect that the high CFR seen in Ugandan cases may result from only the more severe cases presenting to health facilities and being detected by disease surveillance. Most RVF cases may first be confused with malaria, with some patients also having concurrent malaria, only for them to deteriorate clinically with more severe symptoms. Rift Valley fever and other hemorrhagic fevers are usually suspected when patients do not improve with antimalarial treatment and start showing bleeding symptoms, which are typically manifested in the end stage of the disease, when clinical recovery is less likely. The cases in these outbreaks were first detected using passive surveillance; additional cases were identified through active case finding during outbreak investigations. Although the majority of the cases (73%) were detected by passive surveillance, it is important to note that active case finding helped detect 14 additional cases. Active case finding was performed during each outbreak investigation following initial case detections found by passive surveillance, and this should be maintained as common practice. Most confirmed cases (90%) were in adults 18 years and older. We believe this is likely related to specific occupational groups, because RVF tends to be an occupational hazard for certain professions. Adults are more likely to be exposed to infected animals, or their infected body fluids or products through occupations such as abattoir workers, animal health workers, herdsmen, and cattle keeping. In a study conducted in Kabale, Uganda, in 2016, eight individuals younger than 19 years had no detectable antibodies against RVFV. In the reporting period 2017–2020, 92% of all confirmed RVF cases were male who tended to have high-risk occupations in Uganda. Studies in Uganda and other areas in East Africa reinforce the fact that people involved in contact with livestock are at greater risk of contracting RVF.^7^^–^^10^^,^^28^ Most of the patients we identified through surveillance resided within the southwestern districts that make up the cattle corridor of western and central Uganda, representing 93% of all cases. The cattle corridor of Uganda is where a majority of the cattle keeping and commercial livestock trade is carried out, and hence more contact with livestock is likely to occur. Other risk factors for acute RVF positivity in humans include being a butcher or animal slaughterhouse worker and having direct contact with livestock.

We observed that the majority of RVF cases were detected in 2017 and 2018 (Figure 2), following enhanced surveillance for EVD, which was ongoing because of a large outbreak in the neighboring DRC, leading to a high index of suspicion of a VHF. Often, surveillance activities increase after an outbreak of diseases of public health importance, such as VHFs that have clinical characteristics similar to RVF in the early stages of illness onset, as was the case with the EVD outbreak in 2018. However, we did not see this same increase in suspect VHF cases in our surveillance with the COVID-19 outbreak that began in late 2019 and throughout 2020, as the detection of other infectious diseases reduced drastically. This could be related to many factors, including the lockdowns that were instituted to control COVID-19, while at the same time limiting overall health-care access to patients because of restricted movement. We hypothesize that most cases of RVF, and even other more severe diseases, could have gone undetected because of COVID-19 lockdowns, but we cannot quantify the effect of such measures using the large surveillance system. There is a need to discuss surveillance of other equally severe and more infectious diseases with respect to the COVID-19 pandemic and other consequences arising out of these outbreaks.

Clinically, most of the confirmed cases presented with fever (69%) and unexplained bleeding (69%) (Table 2), as well as other nonspecific symptoms, making it difficult to differentiate RVF from other more common infectious diseases such as malaria, typhoid, brucellosis, and other emerging infectious diseases in the tropics. Some patients did not present with fever, especially cases that were detected late in illness onset or had mild, subclinical infections. Therefore, using fever alone as a required element of the VHF case definition for surveillance of RVF may miss true cases. The same applies to cases presenting with bleeding, which is sometimes used by clinicians as the sole indicator of suspect hemorrhagic fever cases. Bleeding does not occur in all cases, hence the need to develop case definitions that are sensitive enough not to miss cases, but also specific to save surveillance resources.

The first cases identified in 2016 were infected by RVFV of the Kenya-2 clade; this clade had been found previously to circulate in Kenya in 2006 and 2007, and in Sudan in 2007 and 2010. With continued surveillance in Uganda, we identified that this clade continues to prevail and circulate in-country. Of particular interest is the identification of Ugandan RVFV K/E strains from 2018 that are distinct from the Kenya-2 clade that has predominated in eastern Africa since 2006. It is currently unclear whether this K/E branch is a historic clade or a newly developed clade that is arising because of a potential antigenic escape in livestock or people (or a result of transmission from a different mosquito vector). The majority of these sequences (four of five sequences) originated in districts from southwestern Uganda in 2018 and could represent a RVFV clade spreading from nearby Rwanda, Tanzania, or DRC. Increased RVFV surveillance in these countries could help shed light on the ancestry of this K/E clade. The relationship between the January 2018 sequence from the Yumbe District (Northwest Uganda) with a sequence originating from Luanda, Angola, in 2016 (a distance of 2,400 km) further highlights the need for increased RVFV surveillance across western, central, and eastern Africa, with special emphasis in countries where RVFV is currently presumed to be nonendemic (i.e., Angola and DRC).

This report is limited only to incident cases of RVF and does not reflect the true burden of RVF in Uganda. We may be detecting only the more severe spectrum of RVF cases in Uganda, which make up a small percentage of the true number of active RVF infections. There is a need to conduct a wider study to describe the burden of RVF in Uganda, and to examine morbidity and mortality by looking at the impact of this disease and how it affects families. This can be combined with a countrywide animal study that also looks at the burden of the disease in humans and animals, as livestock acts as a major carrier and source of infection to humans.

We observed sporadic cases of RVF in Uganda from 2017 to 2020 (*N* = 52), with a high CFR (42%) compared with other East African countries. We also identified contact with animals as the greatest risk factor for RVFV infection. We confirmed the presence of two clades of RVFV circulating in Uganda using next-generation sequencing. This information can be used in designing control and prevention measures against RVF, such as providing health education to groups at risk to encourage the use PPE when handling suspected animals, making sure animal products are well cooked before consumption, using mosquito nets and other insect repellent chemicals to reduce exposure to mosquito vectors, and vaccinating livestock to reduce the potential for human exposure.

## Financial Disclosure

This work was financed through a cooperative agreement between the Uganda Virus Research Institute and the U.S. CDC.

## References

[1] Maes P , 2019. Taxonomy of the order Bunyavirales: second update 2018. Arch Virol 164: 927–941. 30663021 10.1007/s00705-018-04127-3 PMC6581445

[2] Shoemaker T , 2002. Genetic analysis of viruses associated with emergence of Rift Valley Fever in Saudi Arabia and Yemen, 2000–01. Emerg Infect Dis 8: 1415–1420. 12498657 10.3201/eid0812.020195 PMC2738516

[3] Shoemaker TR , 2019. First laboratory-confirmed outbreak of human and animal Rift Valley fever virus in Uganda in 48 years. Am J Trop Med Hyg 100: 659–671. 30675833 10.4269/ajtmh.18-0732 PMC6402942

[4] Adam AA Karsany MS Adam I, 2010. Manifestations of severe Rift Valley fever in Sudan. Int J Infect Dis 14: e179–e180. 19570703 10.1016/j.ijid.2009.03.029

[5] Paweska JT Jansen van Vuren P Swanepoel R, 2007. Validation of an indirect ELISA based on a recombinant nucleocapsid protein of Rift Valley fever virus for the detection of IgG antibody in humans. J Virol Methods 146: 119–124. 17645952 10.1016/j.jviromet.2007.06.006

[6] Diallo M Nabeth P Ba K Sall AA Ba Y Mondo M Girault L Abdalahi MO Mathiot C, 2005. Mosquito vectors of the 1998–1999 outbreak of Rift Valley fever and other arboviruses (Bagaza, Sanar, Wesselsbron and West Nile) in Mauritania and Senegal. Med Vet Entomol 19: 119–126. 15958020 10.1111/j.0269-283X.2005.00564.x

[7] Nanyingi MO Munyua P Kiama SG Muchemi GM Thumbi SM Bitek AO Bett B Muriithi RM Njenga MK, 2015. A systematic review of Rift Valley fever epidemiology 1931–2014. Infect Ecol Epidemiol 5: 28024. 26234531 10.3402/iee.v5.28024 PMC4522434

[8] Nyakarahuka L , 2018. Prevalence and risk factors of Rift Valley fever in humans and animals from Kabale District in southwestern Uganda, 2016. PLoS Negl Trop Dis 12: e0006412. 29723189 10.1371/journal.pntd.0006412 PMC5953497

[9] Sumaye RD Abatih EN Thiry E Amuri M Berkvens D Geubbels E, 2015. Inter-epidemic acquisition of Rift Valley fever virus in humans in Tanzania. PLoS Negl Trop Dis 9: e0003536. 25723502 10.1371/journal.pntd.0003536 PMC4344197

[10] Tigoi C , 2020. High risk for human exposure to Rift Valley fever virus in communities living along livestock movement routes: a cross-sectional survey in Kenya. PLoS Negl Trop Dis 14: e0007979. 32084127 10.1371/journal.pntd.0007979 PMC7055907

[11] Tantely LM Boyer S Fontenille D, 2015. A review of mosquitoes associated with Rift Valley fever virus in Madagascar. Am J Trop Med Hyg 92: 722–729. 25732680 10.4269/ajtmh.14-0421 PMC4385764

[12] Al-Hamdan NA Panackal AA Bassam THA Alrabea A Hazmi MA Mazroa YA Jefri MA Khan AS Ksiazek TG, 2015. The risk of nosocomial transmission of Rift Valley fever. PLoS Negl Trop Dis 9: e0004314. 26694834 10.1371/journal.pntd.0004314 PMC4687845

[13] Shoemaker TR , 2018. Impact of enhanced viral haemorrhagic fever surveillance on outbreak detection and response in Uganda. Lancet Infect Dis 18: 373–375. 29582758 10.1016/S1473-3099(18)30164-6 PMC5894352

[14] Artika IM Ma’roef CN, 2017. Laboratory biosafety for handling emerging viruses. Asian Pac J Trop Biomed 7: 483–491. 32289025 10.1016/j.apjtb.2017.01.020 PMC7103938

[15] Kiyaga C , 2013. Uganda’s new National Laboratory Sample Transport System: a successful model for improving access to diagnostic services for early infant HIV diagnosis and other programs. PLoS One 8: e78609. 24236026 10.1371/journal.pone.0078609 PMC3827263

[16] Bird BH Bawiec DA Ksiazek TG Shoemaker TR Nichol ST, 2007. Highly sensitive and broadly reactive quantitative reverse transcription-PCR assay for high-throughput detection of Rift Valley fever virus. J Clin Microbiol 45: 3506–3513. 17804663 10.1128/JCM.00936-07 PMC2168471

[17] Maurice A de S , 2018. Rift Valley fever: a survey of knowledge, attitudes, and practice of slaughterhouse workers and community members in Kabale District, Uganda. PLoS Negl Trop Dis 12: e0006175. 29505579 10.1371/journal.pntd.0006175 PMC5860784

[18] Schafer IJ Knudsen E McNamara LA Agnihotri S Rollin PE Islam A, 2016. The EpiInfo viral hemorrhagic fever (VHF) application: a resource for outbreak data management and contact tracing in the 2014–2016 West Africa Ebola epidemic. J Infect Dis 214: S122–S136. 27587635 10.1093/infdis/jiw272

[19] Grubaugh ND , 2019. An amplicon-based sequencing framework for accurately measuring intrahost virus diversity using PrimalSeq and iVar. Genome Biol 20: 8. 30621750 10.1186/s13059-018-1618-7 PMC6325816

[20] Grobbelaar AA Weyer J Leman PA Kemp A Paweska JT Swanepoel R, 2011. Molecular epidemiology of Rift Valley fever virus. Emerg Infect Dis 17: 2270–2276. 22172568 10.3201/eid1712.111035 PMC3311189

[21] Samy AM Peterson AT Hall M, 2017. Phylogeography of Rift Valley fever virus in Africa and the Arabian Peninsula. PLoS Negl Trop Dis 11: e0005226. 28068340 10.1371/journal.pntd.0005226 PMC5221768

[22] Nyakarahuka L , 2022. First laboratory confirmation and sequencing of Zaire ebolavirus in Uganda following two independent introductions of cases from the 10th Ebola outbreak in the Democratic Republic of the Congo, June 2019. PLoS Negl Trop Dis 16: e0010205. 35192613 10.1371/journal.pntd.0010205 PMC8896669

[23] Liu J , 2017. The first imported case of Rift Valley fever in China reveals a genetic reassortment of different viral lineages. Emerg Microbes Infect 6: e4. 28096531 10.1038/emi.2016.136 PMC5285499

[24] Henderson BE McCrae AW Kirya BG Ssenkubuge Y Sempala SD, 1972. Arbovirus epizootics involving man, mosquitoes and vertebrates at Lunyo, Uganda 1968. Ann Trop Med Parasitol 66: 343–355. 4404504 10.1080/00034983.1972.11686834

[25] Knust B , 2015. Multidistrict outbreak of Marburg virus disease: Uganda, 2012. J Infect Dis 212 (Suppl 2): S119–S128. 26209681 10.1093/infdis/jiv351 PMC5649344

[26] Nguku PM , 2010. An investigation of a major outbreak of Rift Valley fever in Kenya: 2006–2007. Am J Trop Med Hyg 83: 5–13. 20682900 10.4269/ajtmh.2010.09-0288 PMC2913496

[27] U.S. CDC , 2007. *Rift Valley fever outbreak: Kenya, November 2006–January 2007*. Available at: https://www.cdc.gov/mmwr/preview/mmwrhtml/mm5604a3.htm. Accessed January 27, 2023. 17268404

[28] Swai ES Schoonman L, 2009. Prevalence of Rift Valley fever immunoglobulin G antibody in various occupational groups before the 2007 outbreak in Tanzania. Vector Borne Zoonotic Dis 9: 579–582. 19125662 10.1089/vbz.2008.0108

